# Formative research to scale up a handwashing with soap and water treatment intervention for household members of diarrhea patients in health facilities in Dhaka, Bangladesh (CHoBI7 program)

**DOI:** 10.1186/s12889-020-08727-0

**Published:** 2020-06-01

**Authors:** Elizabeth D. Thomas, Fatema Zohura, M. Tasdik Hasan, Md. Sohel Rana, Alana Teman, Tahmina Parvin, Jahed Masud, Md. Sazzadul Islam Bhuyian, Md. Khobair Hossain, Maynul Hasan, Sanya Tahmina, Farzana Munmun, Md. Abul Hashem Khan, Shirajum Monira, David A. Sack, Elli Leontsini, Peter J. Winch, Munirul Alam, Christine Marie George

**Affiliations:** 1 Department of International Health, Johns Hopkins Bloomberg School of Public Health, Baltimore, MD USA; 2 Infectious Diseases Division, International Centre for Diarrhoeal Disease Research, Bangladesh, Dhaka, Bangladesh; 3 Johns Hopkins Bloomberg School of Public Health, Baltimore, MD USA; 4 Department of Communicable Diseases, Ministry of Health and Family Welfare, Dhaka, Bangladesh; 5 Community Based Health Care, Directorate General of Health Services, Dhaka, Bangladesh; 6 Associate Professor, Department of International Health, Program in Global Disease Epidemiology and Control, Johns Hopkins Bloomberg School of Public Health, 615 N. Wolfe Street, Room E5535, Baltimore, MD 21205-2103 USA

**Keywords:** Diarrhea, Handwashing, Water treatment, Behavior change, Health facilities, Qualitative research, Formative research, Bangladesh

## Abstract

**Background:**

During the time a diarrhea patient presents at a health facility, the household members of the patient are at higher risk of developing diarrheal diseases (> 100 times for cholera) than the general population. The Cholera-Hospital-based-Intervention-for-7-Days (CHoBI7) is a health facility-initiated water treatment and handwashing with soap intervention designed to reduce transmission of diarrheal diseases between patients and their household members. The present research aimed to (1) develop a scalable approach to integrate the CHoBI7 intervention program into services provided at government and private health facilities in Bangladesh; and (2) tailor the intervention program for the household members of all diarrhea patients, irrespective of the etiology of disease.

**Methods:**

We conducted 8 months of formative research, including 60 semi-structured interviews, 2 group discussions, and a pilot study. Thirty-two interviews were conducted with diarrhea patients and their family caregivers, government stakeholders, and health care providers both to explore existing WASH and diarrhea patient care practices in health facilities and to identify considerations for scaling the CHoBI7 program. Fifty-two diarrhea patient households participated in a pilot study of a modified version of the CHoBI7 intervention program for tailoring. Twenty-eight interviews and 2 group discussions were conducted with pilot households to explore experiences with and recommendations for intervention delivery.

**Results:**

The intervention program was modified based on formative research findings. Pilot study participants recognized the benefits of the CHoBI7 intervention program and made suggestions to improve the acceptability and feasibility of the intervention. Modifications included 1) providing additional pictorial modules, cues to action, enabling technologies, and supplies for safe drinking water and handwashing with soap behaviors in the health facility; 2) switching out technology prone to breaks and leaks as well as sourcing plastic technologies from a high-quality, local manufacturer; and 3) including instructions discouraging the non-use or misuse of technologies and supplies. Considerations for scalability include the local availability and marketing of enabling technologies and supplies, staff for program delivery in health facilities, and potential integration into existing government or health promotion programs.

**Conclusions:**

Formative research identified important considerations for the content, delivery, and scalability of the CHoBI7 health facility-initiated WASH intervention program.

## Background

Diarrheal disease remains a leading cause of child death and a major contributor to morbidity across all age groups [1, 2]. During the time a diarrhea patient presents at a health facility for treatment, the household members of the patient are at much higher risk of developing diarrheal diseases (> 100 times for cholera) than the general population [3–7]. This higher risk is likely due to shared, contaminated environmental sources, like household drinking water, and secondary transmission from poor hygiene practices in both the home and health facility, where family members care for patients [8–10]. Access to a safe domestic water source [9] and handwashing with soap [11] have been shown to reduce enteric diseases. However, acceptable and effective interventions tailored to meet the needs of diarrhea patients and their household members, both at a health facility and when they return home, are limited. Health facility-based WASH interventions have been developed in various settings, though few interventions have reported on formative research conducted for intervention development [12–15].

### The Cholera-Hospital-based-Intervention-for-7-Days (CHoBI7)

The original Cholera-Hospital-based-Intervention-for-7-Days (CHoBI7) intervention program was designed for cholera patients and their household members [16]. To target a period of high susceptibility to diarrheal disease [3, 17, 18], the intervention was delivered by health promoters during the seven-day period after the patient was admitted to a health facility for treatment—the high-risk period for the transmission of cholera and other diarrheal diseases [3, 5, 6]. The patient’s hospitalization serves as a disrupting event for the household, creating an opportunity to introduce new behaviors [19]. Household members are both counseled on how to prevent cholera as well as provided with enabling technologies and supplies in order to facilitate behavior change.

The original intervention included a pictorial module (flipbook), delivered at the patient’s bedside, that depicted how cholera spreads and can be prevented. The CHoBI7 moniker is based on the flipbook, as *chobi* means ‘picture’ in Bangla. After the bedside visit, behavioral recommendations were reinforced via daily household visits during the week following admission to the health facility. Enrolled households received an imported, sealed drinking water vessel with tap (Topaz™ [Lion Star Plastic, Jakarta, Indonesia]), chlorine tablets for water treatment, a handwashing station, and a bottle of soapy water (detergent powder mixed with water). Target behaviors included drinking water treatment with chlorine tablets and handwashing with soap at key times (e.g. after using the toilet) during the 7 days following admission to a health facility. Households were counseled to continue protective WASH behaviors after the high-risk period in order to prevent future enteric infections. The original design of the CHoBI7 intervention program was informed by the Risk, Attitudes, Norms, Abilities, and Self-regulation (RANAS) model of behavior change [20] and the Integrated Behavioural Model for Water, Sanitation, and Hygiene (IBM-WASH) [21].

A randomized controlled trial (RCT) of the original CHoBI7 intervention program showed significantly fewer symptomatic cholera infections and a 47% reduction in overall cholera infections among those in the intervention arm versus the control arm [16]. Furthermore, intervention household members had significantly higher instances of handwashing with soap and significantly less *E.coli* in stored drinking water when compared to control households 6–12 months post-intervention delivery [22]. In our assessment of the underlying mechanism of behavior change, response efficacy was associated with habit formation, and disgust, convenience, and cholera awareness were associated with habit maintenance [23]; this points to the importance of considering behavior change theory in the development and implementation of WASH interventions. Interventions informed by behavior change theories that target multiple behavioral determinants are more likely to be successful than interventions that provide information alone [24–26].

### Setting and study rationale

At the request of our partners at the Bangladesh Ministry of Health and Family Welfare, we aimed to develop a scalable approach to integrate the CHoBI7 intervention program into services provided at government and private health facilities in Bangladesh. We also aimed to tailor the intervention program for the household members of *all* diarrhea patients, irrespective of the etiology of disease. To achieve these aims, formative research and the engagement of key stakeholders were required in order to modify the original intervention program. The formative research addressed four research questions: (1) What are existing WASH and diarrhea patient care practices in health facilities? (2) What are households’ experiences with and acceptability of CHoBI7 WASH enabling technology and behavioral recommendations? (3) What are considerations and modifications for taking the intervention program to scale? (4) What modifications to the original CHoBI7 intervention program are needed in order to tailor the intervention for the target population?

**Fig. 1:**
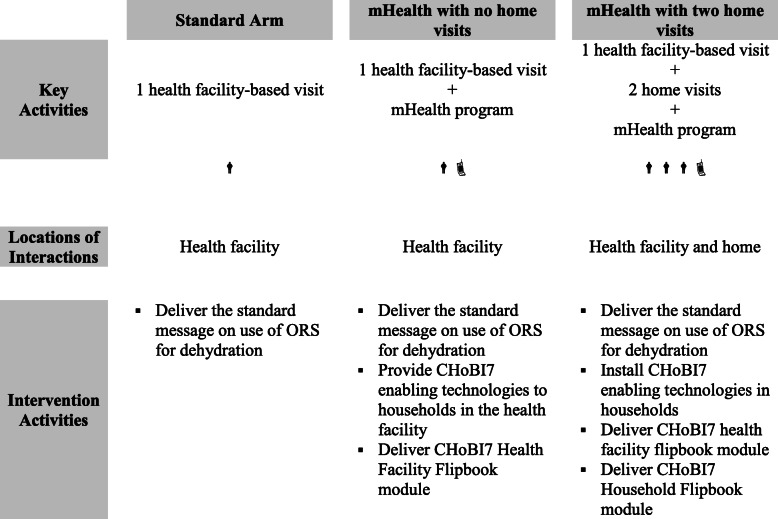
Overview of intervention activities

The goal of the present research was to develop the content and delivery method of a ‘modified’ CHoBI7 intervention program that would require only a single in-person visit in the health facility and could be integrated into services provided to diarrhea patients in health facilities in Bangladesh. Given the objective of developing an intervention program that could be taken to scale, a mobile health (mHealth) program was developed to accompany the health facility-initiated program. Formative research for the development of the CHoBI7 mHealth program is reported elsewhere [27]. The modified intervention was recently evaluated through a three-arm RCT, which took place over 12 months, that compared (1) the standard recommendation to diarrhea patients about oral rehydration solution (ORS) use; (2) health facility initiation of CHoBI7 plus mHealth (mHealth with no home visits); and (3) health facility initiation of CHoBI7 plus two home visits and mHealth (mHealth with home visits) (Fig. 1) (George et al. 2020, submitted).

## Methods

**Fig. 2:**
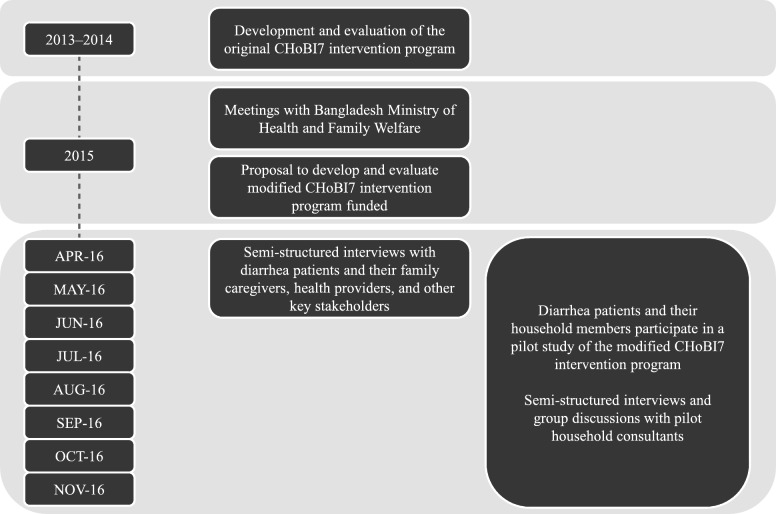
Workflow of research design and formative research activities

Semi-structured interviews and a pilot study for the development of the modified CHoBI7 intervention program were conducted in parallel. An outline of the formative research activities is provided in Fig. 2.

### Data collection

A total of 60 semi-structured interviews were conducted from April to November 2016. Intial semi-structured interviews were conducted with a convenience sample of diarrhea patients (*n* = 5), their family caregivers (*n* = 13), and health providers (*n* = 2) to explore health facility WASH and patient care practices (Table 1). Diarrhea patients and their family caregivers were approached in the hospital, and the interviews took place at the patient’s bedside. Interviews with health providers were arranged over the phone and conducted in the provider’s office. The objective of these interviews was to identify practices and preferences that would necessitate modifications to the original CHoBI7 intervention program.

**Table 1:** Formative research activities

**Diarrhea patient, family caregiver, and health provider interviews**							
• Explore WASH and patient care practices in health facilities to assist with adapting the intervention for delivery in public health facilities		*Diarrhea Patient, Government Hospital*	*Diarrhea Patient, Private Hospital*	*Family Caregiver, Government Hospital*	*Family Caregiver, Private Hospital*	*Health Provider, Government Hospital*	*Total*
	Semi-structured interviews	*4*	*1*	*6*	*7*	*2*	*20*
**Key stakeholder interviews**							
• Address research questions related to scalability of the intervention		*Health-Posting Government Official*		*Senior Scientist*			*Total*
	Semi-structured interviews	*11*		*1*			*12*
**Pilot household consultant interviews and group discussions**							
• Learn about households’ experiences with the intervention and promoted behaviors, and seek advice for how to improve the intervention for the upcoming trial		*Standard Message*		*mHealth with no home visits*		*mHealth with 2 home visits*	*Total*
	Semi-structured interviews	*-*		*17*		*11*	*28*
	Group discussions	*-*		*-*		*2 (19 Participants)*	*2*

Twelve interviews with 10 key stakeholders (1 research scientist and 9 health-posting government officials) were also conducted to explore the scalability of the intervention (Table 1). One government stakeholder was interviewed 3 times. Government stakeholders were purposefully selected to include individuals serving in health services with decision-making power. Appointments for interviews with stakeholders were made over the phone or in-person, and the interviews took place in the interviewee’s office.

From April through November 2016, trained research assistants recruited a convenience sample of 65 diarrhea patients and their household members to participate in a pilot study of the modified CHoBI7 intervention program for continued modification and refinement of the intervention content and delivery. For the pilot study, participants were recruited from 2 participating health facilities: icddr,b’s hospital (private) and a government hospital in Dhaka, Bangladesh. Households were selected for participation if they (1) had a household member admitted to either health facility with three or more loose stools over a 24-h period, (2) had no basin with running water, (3) had a child under 5 years of age living in the home, (4) had at least one active mobile phone (for mHealth message delivery), (5) planned to reside in Dhaka over the following year, and (6) were not involved in another water treatment program. Possession of at least one active mobile phone was required for participation in the pilot study due to the pre-determined inclusion of mHealth as part of the intervention in order to facilitate future scaling up of the intervention program.

Household members of diarrhea patients were eligible for enrollment if their meals were prepared in the same cooking pot as the diarrhea patient for the 3 days prior to the diarrhea patient’s admission to a health facility and if they planned to reside with the diarrhea patient for the next 12 months.

Two households refused to participate after initiating the hospital-based structured observation (activity distinct from the formative research presented here). Eleven households were dropped post-consent either because the study team could not locate the household or the patient left the hospital before receiving the intervention. A total of 52 households completed the pilot study, and 38 households were in the CHoBI7 mHealth arms.

The pilot study design included the three pre-determined arms of the planned RCT (outlined above), with households assigned to either the (1) standard recommendation arm; (2) CHoBI7 mHealth with no home visits arm; or (3) CHoBI7 mHealth with two home visits arm. Intervention arms also received the standard ORS message. While the intervention arms of the pilot study helped to further tailor the intervention program, the standard recommendation arm served as a comparison group to inform operability and is separate from the formative research presented here.

**Fig. 3:**
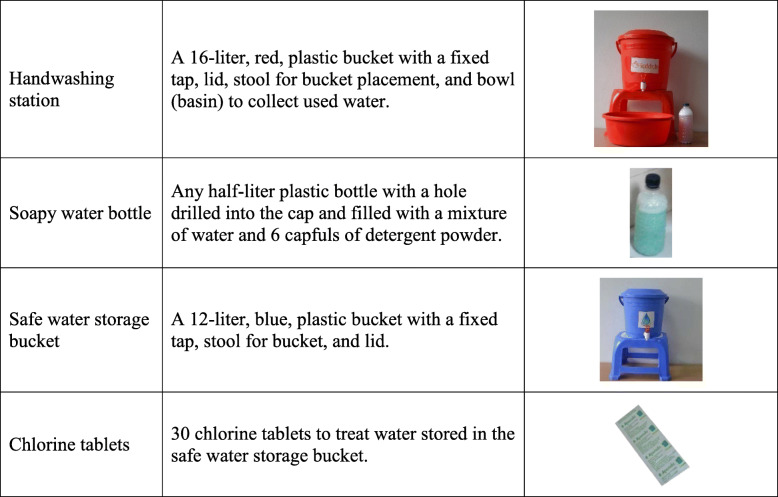
CHoBI7 enabling technology

The modified intervention program included a Health Facility Flipbook about how to prevent diarrhea transmission, which was delivered by a trained health promoter to all intervention households at the patient’s bedside. Households in the mHealth with home visits arm received additional counseling from health promoters using a separate Household Flipbook during two household visits. All intervention households received a handwashing station (a red bucket with tap and lid, a bowl to catch rinse water, and a stool), a soapy water bottle (500 mL bottle, with a pierced lid for dispensing liquid, with detergent powder dissolved in water inside), a safe water storage bucket (a blue bucket with tap and lid and a stool), and a 30-day supply of chlorine tablets for water treatment (Fig. 3). In the mHealth with no home visits arm, the hardware was provided in the hospital; the mHealth with home visits arm received hardware during the first home visit.

Of the 38 households enrolled in the pilot study CHoBI7 intervention arms, 27 households were visited for a follow-up interview (median 6.5 days between enrollment and follow-up) to learn about households’ acceptability of and experiences with the intervention’s enabling technologies and behavioral recommendations, and to seek advice for how to improve the intervention program. One household was visited twice, for a total of 28 pilot study interviews (Table 1). Pilot study participants were called consultants for the CHoBI7 intervention program and were told about their role in an effort to reduce courtesy bias. Follow-up interviews were conducted until we reached saturation.

Two group discussions (*n* = 7 and *n* = 12) were conducted with a convenience sample of pilot study intervention household members to explore the same topics in a group setting and for additional intervention content development. Select findings from initial interviews or early visits to pilot study households were shared and discussed with group discussion participants to facilitate discussion and request feedback on intervention improvement.

Interviews with diarrhea patients and caregivers, health providers, and pilot study participants, as well as the group discussions, were conducted in Bangla by native speakers after extensive training. Ten of the 12 stakeholder interviews were conducted in English and two were conducted in Bangla. All interviews and group discussions were conducted by members of the CHoBI7 research team, who had at least master’s level education in public health or social sciences, after training in qualitative data collection. Interviews and group discussions were done with pre-tested guides, to explore research questions; follow-up probes were used to further explore participants’ responses. Fifty-nine of the 60 interviews and both group discussions were audio recorded. One government stakeholder declined to have their interview recorded. Interviews lasted between 12 min (family caregiver) and 190 min (stakeholder). The group discussions were 127 and 165 min. Extensive field notes were taken by both the interviewer and any present observer to supplement recordings.

### Data analysis

**Fig. 4:**
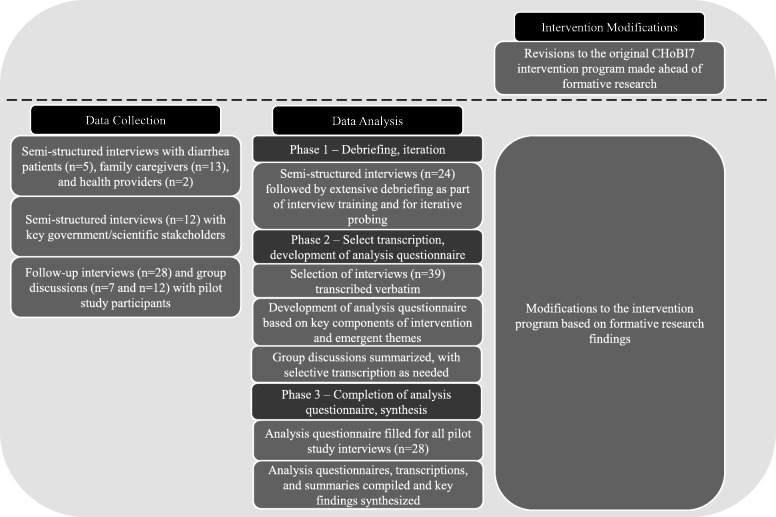
CHoBI7 formative research activities

Analysis of the interviews and group discussions was completed in three phases (Fig. 4). In the first phase, interviews (*n* = 24; 13 diarrhea patient/family caregivers and 11 pilot study) were followed by extensive debriefing to allow for iterative probing on emerging themes [28]. Second, a selection of interviews (*n* = 39; 16 diarrhea patient/household member, 14 pilot study, 9 stakeholder) was transcribed verbatim in the language in which the interviews were conducted. Transcriptions were reviewed by at least one other member of the research team for quality control. Transcriptions were not returned to participants for their review. The transcriptions were then used to develop a summary template or ‘analysis questionnaire,’ based on components of the intervention and emergent themes. Finally, the analysis questionnaire was completed for all pilot study interviews. Group discussions were summarized, and selected quotes were transcribed. Field notes further supplemented transcriptions and summaries. Completed analysis questionnaires, transcriptions, and group discussion summaries were reviewed to identify and compile key findings and quotes for each intervention component. Based on formative research findings, modifications to the CHoBI7 intervention program were made throughout the data collection and analysis process.

### Data synthesis

For the final synthesis of findings, contextual, psychosocial, and technological factors that emerged from formative research were organized using the IBM-WASH framework.

## Results

Findings are presented by research question and include insights into WASH and diarrhea patient care practices in health facilities, pilot study feedback on enabling technologies and behavioral recommendations, and considerations for scalability that emerged from discussions with key stakeholders.

### What are existing WASH and diarrhea patient care practices in health facilities?

Treated drinking water (filtration) was available for consumption at icddr,b but not at the government facility. However, in both health facilities, patients and their family members often brought their own drinking water, such as purchased bottled water or boiled water from home. One health provider at the government facility specified that family members were counseled to bring in boiled water for patients because the quality of water at the facility could not be guaranteed. At icddr,b, one caregiver cited crowding near the facility’s water filter and the filter’s slow flow rate as reasons why he purchased bottled water during his family’s stay.

Soap was also brought by patients’ families. Icddr,b provided liquid soap at sinks, though participants said not all sinks had soap. Some families brought in soap or detergent for personal use, such as to wash *kanthas* (‘blankets’) used by patients. Although handwashing facilities at icddr,b were considered satisfactory, caregivers still reported barriers to handwashing:

“… Here [icddr,b], the mother can only stay with the patient, so I will take care of the baby or myself. That’s why [handwashing is] not done properly. But, in [the] home, if someone helps, then we can take care [of the child] properly. Now, here, do I have to take care of the baby or wash hands? … If someone is with me, then I can keep my child neat and clean. Here it is not possible.”

At the government facility, handwashing and toilet facilities were considered poor by patients and providers. One patient reported visiting the toilet and not being able to wash his hands due to lack of soap. Project staff found that hardware on sinks adjacent to the diarrhea ward was broken or missing. One health provider noted that they did not have a sufficient soap supply to meet handwashing needs, and they had never visited the facilities for the patients.

Health facilities limit the number of caregivers that can attend a patient. Some mothers said that they could not leave to wash their hands or relieve themselves for fear that their child would roll off the bed in their absence. Another concern was that a child might defecate while the caregiver was being served food. Indeed, interviewers noted missed opportunities for handwashing during interviews, sometimes because the patient required care at that same moment.

Patients and their family caregivers were asked whether anyone from the health facility had provided health-related information. One participant at icddr,b said that a nurse had discussed saline intake and iron supplements. Another patient said that no one had been by because the staff were too busy and that he preferred to discuss health issues with family. Health providers at the government facility said that patients were given hygiene recommendations upon discharge, which may explain why, during our interviews (before discharge), very few participants were able to recall their receiving health advice from health facility staff.

### What are households’ experiences with and acceptability of CHoBI7 WASH enabling technology and behavioral recommendations?

#### Chlorine tablets for water treatment

Pilot participants reported various household water treatment practices prior to enrollment into the pilot study, including consumption of *kaccha pani* (‘raw, untreated water’) from municipal water pumps or tube wells and filtering and/or boiling water. For most, use of chlorine for water treatment was a new practice, though some participants were familiar with a local water purification tablet called Halotab®. Most participants after receiving CHoBI7 intervention delivery in the health facility were able to describe the proper use of the intervention-provided Aquatabs® (chlorine tablets) for water treatment. However, some participants struggled to recount the correct water-to-tablet ratio or the length of time water could remain safe for consumption after treatment. A few participants expressed concern for human error, such as taking the tablets as medicine, even though the flipbooks included a pictorial warning to not ingest the chlorine tablets directly. Still, the chlorine tablets were often referred to as *aushoodh* (‘medicine’). One patient attributed his recovery to the chlorine tablets:

“I’m good now only [because of] this chlorine tablet. If I didn’t get this tablet, I would not have been healthy.”

Discussions about the chlorine tablets often turned to the taste and smell of chlorine-treated water. Participants said treated water smelled or tasted like “gas” or “medicine.” Though most participants mentioned taste as a concern, many said they were able to overcome it within a few days:

“If they drink this water one or two days, they will get used to it … Perhaps they would feel bad [for the] first one or two days, [but] they will be okay after that. I felt bad [for the] first few days, but now I got used to [it].”

One participant said she had served the chlorine-treated water to employees in her shop, and they liked it. However, some households said they stopped using chlorine-treated water shortly after returning home from the health facility, citing aversion to taste or smell, concern that the water was not safe for consumption, or an upset stomach as reasons for discontinuation.

For those who did regularly use the chlorine tablets, the convenience of the tablet compared to boiling drinking water was frequently mentioned:

“[The] tablet is a shortcut system and more convenient ... it takes only 30 minutes. Boiling needs more than 30 minutes … Sometimes there is no gas supply for the stoves, or sometimes the supply water is too smelly and dirty to boil it. That’s why I think tablets are better.”

Challenges with boiling water, such as restricted stove access, gas supply, or time limitations, made chlorination an appealing alternative. That said, most participants said they would boil to treat water after the 30-day supply of chlorine tablets ran out. Several individuals said they looked for chlorine tablets in the market; others said it would be best if they were given more tablets. We recommended using chlorine-treated water to wash fruits and vegetables eaten raw, but one participant said this would be a waste of treated water.

#### Safe water storage

At the time of the interviews, most participants were using the intervention-provided blue bucket with tap for storage of either chlorine-treated or boiled water for drinking. Participants expressed appreciation for the functionality and convenience of the safe water storage bucket. For the most part, the bucket was considered good quality and durable. Several participants preferred a larger bucket (e.g. 20l) to accommodate drinking water needs, though one participant noted that people may not be able to lift buckets if they were larger, and another said that the 10l size was best to help with measuring water amounts to be treated with chlorine tablets.

Households reported using a traditional water storage vessel, or *kolsi*, either alongside or prior to the receipt of the safe water storage bucket. A preference for the plastic bucket over the metal *kolsi* was common; one reason was because plastic would not rust. Both the lid and tap were also important features of the bucket:

“A tap is attached to this [bucket]. If it were a normal bucket, [people] would say, ‘Why will we use this bucket instead of a *kolsi*? [The] *kolsi* is perfect for me.’ But, since this bucket has a tap, I can take water from this bucket through this tap. It is a shortcut with less trouble.”

The lid was seen as a means to keep dirt and hands out of drinking water, something that *kolsis* did not always have. One concern about the tap was durability, as some taps initially leaked. One participant did not think she could replace the tap if she needed to buy a new bucket, saying she would need to return to dipping her hand in the bucket with a cup. Another said she would pay 200–300 BDT (3.50 USD) for a new tap because she was accustomed to it now. The actual cost of the tap in the local market is 25–40 BDT (0.30–0.48 USD). One suggestion given by a participant was to fix the tap one inch higher to make it less likely to break. Another issue was that children would open the tap and spill/waste water; one participant stored her drinking water in plastic bottles as a result. On the other hand, the tap was also seen as a means for children to access safe drinking water:

“I like this bucket because it has a tap. My kids couldn’t pour water from the storage [container] before, but now they don’t have to pour it. They can just open the tap and drink from it using a mug.”

Interestingly, three participants mentioned plastic quality, naming the company (a popular plastics company in Bangladesh) where project buckets were sourced by name and saying that this plastic “did not harbor germs” or “had no typical plastic smell.” One maintenance issue mentioned was that the bucket would sometimes acquire a “slimy layer” when water was kept in it for too long, making regular cleaning and drying of the bucket necessary. Two participants mentioned leaving their buckets with relatives; one reason given was lack of space.

#### Handwashing station and soapy water bottle

Many discussions about the handwashing station (water storage bucket with tap, bowl to catch rinse water, stool to elevate bucket, and soapy water bottle) began with a recitation of the key times for handwashing promoted in the flipbooks. Several households reported that the frequency of handwashing in the family had increased since intervention delivery:

“We used to wash our hands after coming from the toilet and going out for a walk. After coming from the walk, we used to eat and feed our children without washing hands. But, now, after coming from outside, we wash our hands and then feed the children. The water and the soap are here.”

As noted, one reason mentioned for the increase in handwashing was that “everything was in one place.” The handwashing station served as a facilitator and a reminder to wash hands with soap. A key difference from typical practice was that handwashing inside or near the house was made possible by the handwashing station:

“There’s a bucket, a bowl, and soap there [at the household entrance]. We wash our hands easily with soap there after coming from outside. But, if there is no such arrangement, we have to go to the tube well and press it for water. Moreover, there are women there. Men do not always want to go there in the middle of the women.” (Female participant).

The need to visit a water pump to wash hands was viewed as an inconvenience and barrier to handwashing immediately following a contaminating event (e.g. using the toilet); pumps were reported to be crowded and required the use of communal bar soap, which several participants considered unhygienic:

“In the case of bar soap, many people use the same soap; germs and disease spread in this way. I think the soapy water is better.”

One participant stated that the soapy water was the “most useful” CHoBI7 intervention component. Soapy water was considered easy to make, affordable, and to produce a better lather compared to bar soap. Participants said neighbors or relatives inquired about soapy water and later made it themselves. That said, one participant did not believe soapy water could remove germs, stating “After all, it is laundry detergent.” Another participant said the soapy water caused a “burning sensation” on her hands.

When asked what challenges could come between handwashing with soap at key times, participants said that “laziness,” “busyness,” and the distraction of children were barriers. Participants also said project staff had motivated household members, and it was now up to individuals to adopt the recommended behaviors. Additional recommendations included promoting regular nail-cutting and having multiple handwashing stations (e.g. one for inside and one near the kitchen). Similar to the safe water storage bucket, young children would play with and spill the handwashing water and soapy water. One mother said she had to keep the bowl stored away when it was not in use because she was concerned that her child would play in the discarded handwashing water and become ill.

The stool was seen as a valuable part of the station:

“Since I have this stool, I can easily take water from [the handwashing station]. It would be difficult to take water if there was no stool—our hands might touch the ground while washing them. Germs could come to our hands from the ground.”

Participants also stated that some of the handwashing stations were leaking. Other challenges included breaking the bowl and displacement of the tap. Two group discussion participants mentioned trying to fix the issues by themselves but needing to seek assistance.

The color distinction between the safe water storage bucket (blue) and handwashing station bucket (red) was considered helpful for separating “water for different uses,” with someone mentioning that red indicated the bucket was not for drinking, like the red-marked tube wells that indicated arsenic:

“The color of the buckets is different, this is good. It helps to determine that this bucket is for drinking water and this one is for handwashing.”

However, one group discussion participant said that people may be confused and think that the water in the red bucket *contained* arsenic.

Finally, one participant reported that her neighbors had started to use a bucket like hers to wash their hands “so they could live well and not need to go to the cholera hospital.”

#### Cue cards and flipbooks

For the most part, participants felt the cue card design did not require modifications. Several commented on the importance of the pictures on the cards to help illiterate participants or children understand behavioral recommendations. One participant said the cue cards were superior to oral instruction. However, others said that people would not understand the cards without an accompanying explanation.

The cue cards were considered important reminders in case household members forgot the key times for handwashing with soap or how to use chlorine tablets. One participant referred to the cue cards as “friends” because they helped her stay healthy:

“These [cards] are like our friends; they are keeping us away from diseases, protecting us.”

Hanging the cue cards in sight or adjacent to the related behavior (e.g. hanging the chlorine treatment cue card next to the safe water storage bucket) was recommended by participants. One participant recommended hanging cue cards outside so that others could see the cards and learn from them. A few participants reported not hanging the cards due to busyness or lack of space. One participant commented on the absence of repair/maintenance information on enabling technologies in the flipbooks, pointing to a concern about guidance for how to resolve such issues.

### What are considerations and modifications for taking the intervention program to scale?

#### Enabling technologies and supplies

Government stakeholders had experience with the aforementioned Halotab® tablets, distributed in the past by the government during outbreaks or national emergencies (e.g. floods). Stakeholders were concerned about the population’s acceptance of the tablet due to its taste and worried that the tablet might be misused as medicine. One government stakeholder suggested incorporating flavors, like strawberry, to the tablets to improve the taste. Important to note is that Halotab® is no longer available on the local market. Some government stakeholders advised us to partner with local pharmaceutical companies for marketing chlorine tablets and to improve the taste of these tablets. A concern was that local pharmaceutical companies would not develop an alternative to Halotab® due to low anticipated demand and earnings. One stakeholder suggested a partnership with Non-Governmental Organizations (NGOs) to distribute Aquatabs® to health facilities in the future, thereby avoiding the government-mandated tendering process.

In our discussions on hand hygiene, one stakeholder noted that the current government position on hygiene was that it was the responsibility of the individual:

“… Hygiene … is now taken by [the] government as ‘one individual, one hygiene.’ Your hygiene, it will be by your own way … Hygiene is now considered the individual level, not the state level …”.

The suggestion here was that hygiene interventions needed to be sustainable without long-term support of the government (e.g. continued provision of soap). Both liquid soap and hand sanitizer were said to be supplied in government health facilities, though for health professionals, only. To the stakeholders we interviewed, using detergent to wash hands was a novel concept that aligned with an ongoing government subsidy program by the Government to keep costs of detergent low:

“Detergent in Bangladesh is now very cheap. That is the government action. And people can buy [it] at low cost and can use [it].”

The same individual was surprised to learn that soapy water was made using the very same ‘non-deluxe’ (i.e. ordinary) detergent subsidized by the government. One stakeholder recommended we explore other detergents being used in the home to be sure they were suitable for soapy water and to keep in mind the growing use of liquid soap in households.

#### Mass media for promoting CHoBI7 enabling technologies

The majority of government stakeholders said it would be difficult to provide a handwashing station and drinking water container to all diarrhea patients in health facilities in Bangladesh. They recommended ensuring items were available in the market for people to buy and promoting them with a marketing campaign. Mass media was considered the best avenue to promote enabling technologies to households. One recommendation was to partner with major plastics companies in Bangladesh to market products using mass media and to make the handwashing station and drinking water vessel with tap as attractive as possible for marketing:

“They [the patients] have money to purchase this hardware, but this hardware should be available … You motivate people to produce this type of hardware and they will popularize this hardware to the people through electronic media.”

“The manufacturer should be responsible [for messaging] because [the hardware] is a commercial project ... we should talk with the manufacturer, convince them … if you develop in this way, and sell it [for] 130 Taka, so you are getting a profit [of] 32 Taka. That will give the opportunity to the manufacturer to make … more profit … If you give the right messages in the advertisement—that is we’ll ensure you the … safe drinking water...people [will] buy it.”

Television was the communication channel most recommended for delivering CHoBI7 behavioral recommendations. Radio, newspapers, and electronic media were also mentioned. It was recommended that we develop up to five short commercials, and to show these regularly through Bangladesh television channels. A scrolling message on the television was also suggested. The success of companies like Lifebouy (a ‘health soap’ marketed by Unilever) was mentioned as a potential model for CHoBI7, with one stakeholder calling Lifebuoy the “grand show for handwashing.” Mass media campaigns were also encouraged for the marketing of chlorine tablets.

#### Delivering the CHoBI7 intervention program in health facilities

Stakeholders suggested that the scaling up of the CHoBI7 intervention program could involve health educators in government *upazila* (‘sub-district’) health complexes or community health providers at community clinics. Community clinics were said to have high coverage across the country; however, their health providers are known to have high workloads. One respondent recommended delivering the program at community clinics and hiring individuals to deliver the intervention. Health educators were considered to have the advantage of being located in *upazila* health complexes, where patients often stop first before going to community clinics.

A major challenge is the absence of community clinics and sufficient health educators in urban Bangladesh. For urban settings, it was recommended that we partner with local NGOs, urban health centers, government dispensaries, Dhaka Water Supply and Sewerage Authority, the Local Government Engineering Department, and Dhaka City Corporation to deliver the intervention program. Providing the handwashing station and safe water storage bucket directly to diarrhea patients was not considered a practical option for future scaling up of the intervention; one government stakeholder said that, if we gave diarrhea patients these items, the inventory would be exhausted within a month because individuals without diarrhea would come to facilities to collect them. One suggestion was for a demonstration handwashing station and drinking water container to be on display in health facilities for patients and family members to use as well as for health providers to recommend patients and their families buy these items after discharge:

“If you give … these types of accessories [handwashing station and sealed water vessel] to the [health facility] ward … and if it is available in the market, then they can tell the patients that, yes, now you’re using it, go home and buy it and use it.”

There was general agreement that individuals would be willing to buy the enabling technologies. It was also noted that many households already had these items in their homes (e.g. bucket, stool, basin) but were not using them for WASH purposes.

Government stakeholders recommended that doctors, nurses, and health facility support staff be trained to deliver the CHoBI7 intervention program:

“It will be more effective if you train our health educator(s), because we have health educator(s) in every district and every *upazila* health complex … Every day, they [will] give [the] message to the outpatient(s) or people coming in the hospitals … First you have to train them … as well as the doctors and the supporting staff also … and you put this hardware set, one set, in every hospital.”

The ‘diarrhea corner’ or ‘ORT (Oral Rehydration Therapy) corner’ present at many health facilities, where the use of ORS is promoted, was mentioned as an ideal location for delivering an intervention. It was recommended that the Health Facility Flipbook be delivered to both admitted and outpatient diarrhea cases and that posters or leaflets be given to patients.

Several government stakeholders mentioned that there would be room for the CHoBI7 intervention program in upcoming, already-funded initiatives. Others said that, for the intervention to be taken to scale, funding would need to be secured. Government stakeholders recommended CHoBI7 be part of the upcoming National Operational Plan for Communicable Diseases Control (CDC) Department:

“If you include this [the CHoBI7 program] in the CDC operational plan to train up the health educators … the Health Education Bureau, they will include this soap [and] hand washing [recommendations] ... they [will] include these activities to train-up five hundred to one thousand people.”

Integration of the CHoBI7 intervention program with existing government programs, such as a weekly health awareness program delivered at *upazila* health complexes, was mentioned as a method to minimize cost. It was also recommended that WASH behavioral recommendation development be done through the Health Education Bureau. One individual stated that, before the program could be recommended for scaled-up delivery, evidence that the intervention was effective would need to be published.

### What modifications to the original CHoBI7 intervention program are needed to tailor the intervention for the target population?

In preparation for the formative research presented here, revisions to the original CHoBI7 intervention program, enabling technologies, and behavior change communication plan were made ahead of initiating the research activities presented here. These updates were based on experiential knowledge of the study setting, previous formative research, and pre-determined changes in program content (i.e. focus on all diarrhea etiologies). For example, the wording of the flipbooks was updated to focus on all types of diarrhea rather than just on cholera. Data collection to inform the development of the scalable approach to CHoBI7 was initiated after these preliminary updates.

The following additional modifications were made to the intervention program based on formative research findings both to tailor the intervention to target households as well as to address considerations for scalability (Table 2).

**Table 2:** Modifications to the original intervention package based on formative research findings, and considerations for scalability

Original CHoBI7 Intervention Design	Formative Research Finding	Modified Intervention Design	Considerations for Scalability
Health facility-initiated intervention delivered by health promoters to cholera patients and their household contacts over the 7-day high-risk period for transmission of disease to household members.		Health facility-initiated intervention delivered by health promoters to diarrhea patients and their household contacts in the health facility over the 7-day high-risk period following admission to the health facility.	Replacement for study-supported health promoters (e.g. health educators or nurses). Utility of mass media channels to alleviate burden of health communication at the health facility. Cost of program materials provided at scale. Market availability of promoted enabling technologies and supplies (e.g. handwashing station, safe water storage bucket, and chlorine tablets) Integration of intervention program into existing government health programs.
Pictorial module on how cholera spreads and can be prevented, delivered by a health promoter at the patient’s bedside.		Pictorial module on how diarrhea spreads and can be prevented, delivered by a health promoter at the patient’s bedside.	
Communication module reinforced during daily household visits during the one-week intervention period.		Mobile messages reinforced either through only voice and text messages weekly for 1 year (mobile health (mHealth) with no home visits), or mHealth and two home visits.	
Imported, sealed drinking water vessel.	Importance of using locally available items to maximize sustainability and keep cost of intervention low. Need for a durable tap. Importance of elevating water storage container from the ground for hygiene purposes. Absence of reliable potable water in some health facilities. Recommendation (or preference) to bring in own source of drinking water for health facility stay. Residue build-up in safe water storage bucket.	Locally made plastic bucket with lid and added durable tap. Provision of a stool to elevate bucket. Provision of chlorine tablets and one bottle of chlorine treated water for health facility stay. Messaging around cleaning the safe water storage bucket regularly to avoid build-up related to long-term water storage.	Low-cost, durable, locally made option will need to be made available on the market. Availability of potable water in health facilities.
Chlorine tablets for water treatment	Dislike of bitter taste of chlorinated water. Barriers to regular boiling of water. Absence of safe drinking water source in health facility, or practice of bringing in own drinking water. Trouble distinguishing chlorine as ‘water treatment agent’ rather than ‘medicine’.	Chlorine tablets for water treatment. Messaging relating the taste of chlorine to local medicinal plants and bitter foods with health benefits. Messaging around chlorine as water treatment, not curative medicine. Taste tests of chlorinated water while in the health facility. Messaging on boiling water after the 30-day supply of chlorine tablets is exhausted.	Low-cost, locally made option will need to be available via Bangladesh-based pharmaceutical companies
Handwashing station	Handwashing aid needed at bedside. Misuse of enabling technologies. Use of handwashing station by children.	Provision of bottle of soapy water bottle for duration of health facility stay. Additional information in the communication module around importance of enabling technologies for a safe and healthy environment to avoid misuse. Additional messaging around teaching children how to use enabling technologies.	Low-cost, durable, locally made option will need to be made available on the market.
Bottle of soapy water	Need for healthy lather from soapy water without drying of hands. Need for multiple soapy water bottles.	Increased the ratio from 4 capfuls per 500 mL to 6 capfuls per 500 mL. Recommended households make multiple soapy water bottles for placement in home, latrine, and kitchen areas.	Current Government detergent subsidies likely to support soapy water as a low-cost alternative to bar or liquid soap.

#### Chlorine tablets for water treatment

Given that the taste of water treated with chlorine was a concern expressed by most, a comparison of chlorine to local foods and plants that are health-protective but have a bitter taste (e.g. *neem* plant and bitter gourd) was added to the flipbooks [29]. In addition, during intervention delivery in health facilities, all family caregivers present were given a taste of chlorine-treated water and were probed to discuss their concerns, giving health promoters the opportunity to counsel them on the taste. Based on the finding that many patients and household members thought that chlorine tablets were curative, participants were reminded that the tablets were not medicine but were a method of water treatment. Household members were also counseled on how to make the transition from chlorine-treated water to boiled water after the 30-day supply of tablets was finished.

#### Safe water storage

The original CHoBI7 intervention program had included an imported safe water storage container. During piloting, project staff met with local plastic suppliers to select a durable plastic bucket option for the safe water storage bucket and handwashing station and their accompanying stools. Based on pilot household feedback, the initial taps for both buckets were switched out early on in the pilot for a sturdier option, and extra washers were added to limit the possibility of leaks. Given households’ concern for build-up on the bucket related to long-term water storage, we also included a recommendation to clean the bucket once per week. Initially, we considered recommending that all patients keep their safe water storage bucket in the health facility, but we found that limited space and sometimes-soiled floors made use of the bucket in the health facility for drinking water a challenge, even with the intervention provided stool. Following the finding that family caregivers and patients often brought their own water to the health facility during their stay, we decided to leave a bottle of chlorine-treated water for them to use during the remainder of their stay.

#### Handwashing station and soapy water bottle

Interviewers’ observations and interviews with patients, family caregivers, and health professionals necessitated handwashing technology at the patient’s bedside. We tried multiple modifications to the intervention during the pilot study period to facilitate health facility-based handwashing with soap. Ultimately, we decided to recommend that all intervention patients and caregivers keep a soapy water bottle and a separate bottle of water for rinsing to facilitate handwashing while in the health facility. We recommended that caregivers wash their hands using the soapy water over the plastic basins that were often available for storage and/or trash. In the mHealth with no home visits arm, households were given the handwashing station in the health facility since no subsequent home visits were conducted to provide intervention materials. Promoters asked patients and caregivers to use their own detergent powder for preparing soapy water, as many had brought this with them to the health facility. If they did not have their own detergent powder, we provided it.

**Fig. 5:**
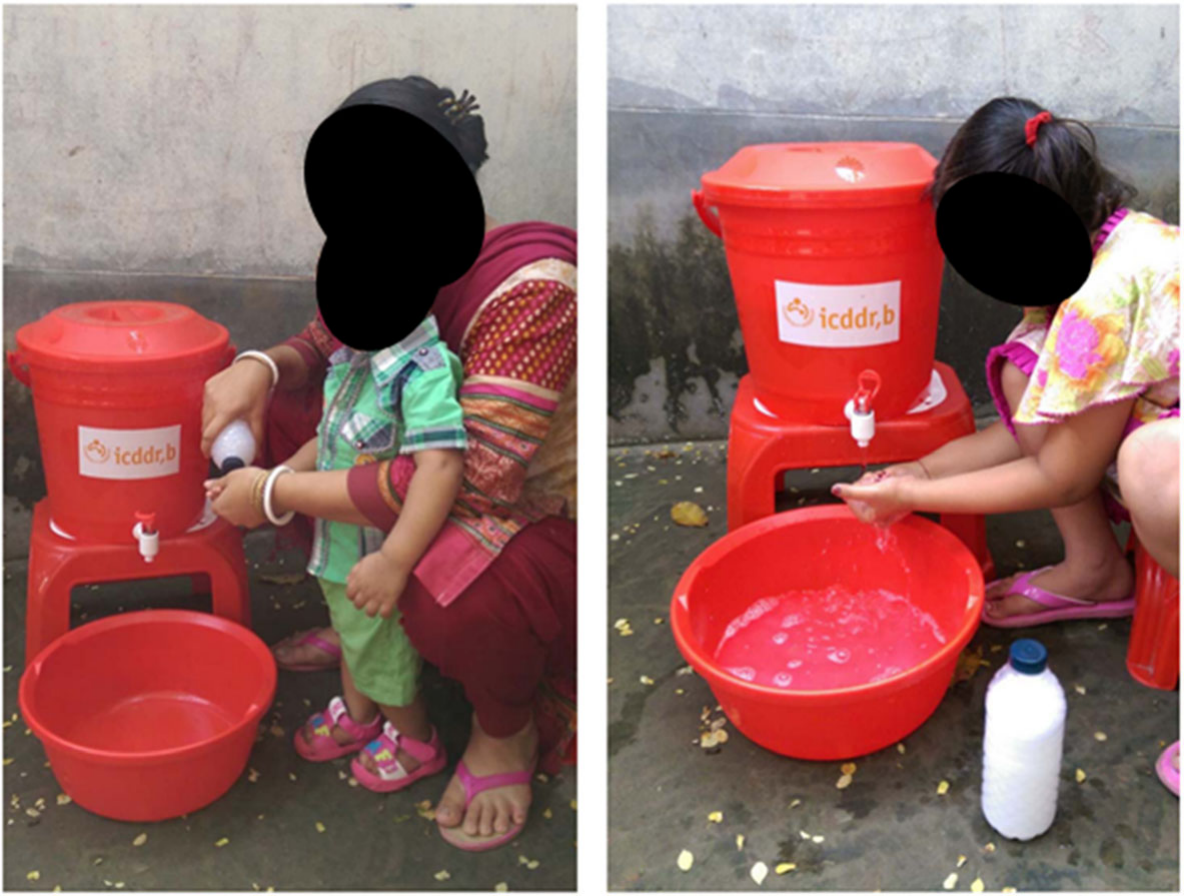
Flipbook modification to include information on caregiver-assisted handwashing with soap for younger children

Once individuals returned home, we encouraged households to make multiple soapy water bottles to facilitate handwashing at key times at different locations (e.g. latrine and kitchen areas). We also encouraged household members that left the house during the day to take a soapy water bottle with them to facilitate handwashing while away from home. We modified the detergent-to-water ratio for soapy water, increasing the ratio from four to six capfuls of detergent powder per 500 mL of water to ensure that a healthy lather was produced during handwashing. Following feedback about children not using the handwashing station, we added images and recommendations about the importance of teaching children how to use the handwashing station properly as they grew, emphasizing caregiver-assisted handwashing with soap for younger children and independent handwashing with soap for older children (Fig. 5).

Given the misuse of enabling technologies observed during the pilot study period, we emphasized the importance of using each item, as directed, for a safe and healthy home environment. Comical photos of technology misuse, including using the stool as a television stand and using the basin as a place for dirty dishes or clothing, were shown by the health promoter in the health facility, and misuse was discouraged.

#### Flipbook and cue cards

In the initial pilot rollout, there were six accompanying cue cards. Study staff deemed six too many because all available spaces on the walls in households were being covered with project materials. As a result, we reduced the number of cue cards from six to four. We also developed a sticker with a picture of a model family drinking water next to the safe water storage bucket and the accompanying message: “Germ free hands, safe water, make me healthy.” We encouraged households to place the sticker on our intervention items, mirrors, or elsewhere in the home as a reminder to practice the behavioral recommendations.

### Synthesis of findings: IBM-WASH factors of influence for the CHoBI7 intervention program

In Table 3, we organized the contextual, psychosocial, and technological factors identified from this formative research according to the IBM-WASH framework [21]. We were able to identify and modify multiple factors of influence through the formative research activities presented here, and organizing them with IBM-WASH helps to acknowledge the multiple levels and dimensions that influence WASH-related behaviors in this context. For example, interviews with diarrhea patients and their family caregivers, as well as with health care providers, provided important insights into the health facility environment (IBM-WASH community-level, contextual and technological dimensions), helping to highlight access-related barriers to the CHoBI7 behavioral recommendations that necessitated modifications to the intervention (e.g. provision of soapy water bottles in the hospital [Tables 2 and 3]). Pilot study participants’ insistence that individuals would become accustomed to the taste of chlorinated water over time (IBM-WASH individual-level, technological dimension) led us to front-load behavioral recommendations to help participants through their initial dislike of the taste of chlorinated water. We also realized that, as households finished their supply of chlorine tablets and needed to adopt boiling, we would need supportive mHealth messaging during the transition to alternative methods of water treatment that addressed identified constraints at the IBM-WASH household level. Finally, we identified several higher-level contextual and technological factors that are typically out of the scope of WASH interventions but should be addressed or considered for scale-up, such as pharmaceutical company development and provision of an affordable, locally-available chlorine tablet.

**Table 3:** Key factors related to intervention components as organized by IBM-WASH

	Contextual factors	Psychosocial factors	Technology factors
**Structural**	Government of Bangladesh mandated tendering process for procurement of goods (e.g. chlorine tablets, enabling technologies, etc.). Government of Bangladesh policy that hygiene is the responsibility of the individual.	Government stakeholder interest in, and prioritization of, a health facility-based WASH intervention.	Past use of chlorine tablets by Bangladesh government during cholera outbreaks or high-disease-risk events (e.g. floods). Pharmaceutical company development and provision of an affordable, locally-available chlorine tablet. Plastics company development and provision of an affordable, locally-available handwashing station and safe water storage bucket. Government subsidization of laundry detergent.
**Health Facility/ Community**	Access to/Condition of latrines, basin, potable water, stove use/fuel in the compound or community. Access to/Condition of latrines, basin, potable water in public/private health facilities. Access to markets and market goods (e.g. soap or detergent). Public/private health facility resources and regulations. Informal providers as initial point of treatment.	Prevalence and expectation of promoted behaviors within the local and larger community.	Position of cue cards to increase visibility of key messages for neighbors of enrolled households.
**Household**	Access to/Condition of, basin, potable water, stove use/fuel in the household. Responsibility for water collection, treatment, and storage. Responsibility for children under five years of age. Available space in home for handwashing station and safe water storage bucket. Household size.	Desire to develop and maintain a safe and hygienic household environment.	Modelling handwashing station use for children to reduce non-use or misuse..
**Individual**	Wealth, education, gender, livelihood of primary taker of children under five and primary decision-maker. Age of children in household.	Awareness of diarrheal disease transmission within the household/compound, and high perceived susceptibility of disease. High perceived benefits of adopting protective water, sanitation, and hygiene behaviors. Disgust reaction in response to the information that diarrheal disease can be a result of consuming water or food contaminated with feces. Response efficacy that practice of recommended WASH behaviors will reduce risk of diarrheal disease.	Dislike of taste/smell of chlorine-treated water during. High perceived convenience of handwashing station, soapy water bottle, safe water storage bucket, and chlorine tablets compared to other options. Strengths/weaknesses of enabling technologies for users (e.g. size).
**Habitual**	Environment allows for habit formation and limits habit disruptions.	Existing water collection, treatment and storage practices. Existing hand hygiene practices.	Ease of routine use of handwashing station and safe water storage bucket. Visible handwashing station, safe water storage bucket, and cue cards to serve as cues to action.

## Discussion

Formative research was critical for tailoring the CHoBI7 intervention program to our target population and for identifying approaches for scalable program delivery. Interviews with diarrhea patients and their family caregivers, government officials, and health providers, in addition to the pilot study of the intervention, informed our understanding of factors that are important for a household’s adoption and maintenance of WASH behaviors, modifications needing to be made to intervention program materials, and considerations for delivering the CHoBI7 intervention program in public and private healthcare facilities in Bangladesh. With respect to existing WASH and diarrhea patient care practices in health facilities, interviews provided important insights into the health facility environment, including insights into gaps in diarrhea patient and family caregiver access to water and hygiene while in health facilities that could be filled by the CHoBI7 intervention program.

Pilot study feedback on intervention components was consistent with findings from other settings. For example, the role of taste with respect to acceptance of chlorine treated water has been well documented [18], and our modifications to the intervention to mitigate this concern could be applied in other contexts where treated water is part of an intervention. Similar to other research in Bangladesh, when interviewing pilot study participants, we found that having a handwashing station in the home facilitates handwashing by having all materials required for handwashing in one place; however, durability of the material, cost associated with alternative handwashing agents (e.g. low cost of laundry detergent), and disgust at sharing soap also play a role in whether or not the technology will be used [30]. Exploring households’ experiences with and acceptability of CHoBI7 WASH enabling technology and behavioral recommendations allowed us to tailor the intervention to a specific population.

Interviews with government stakeholders provided valuable insights for scaling up of the CHoBI7 intervention program in Bangladesh. We learned that delivering CHoBI7 enabling technologies to all diarrhea patients in health facilities would likely not be feasible at scale. However, stakeholders recommended promising alternatives, including partnering with plastics and pharmaceutical companies to market enabling technologies and supplies through mass media. These partnerships should be pursued during the transition to scale. The successful scale-up campaign for zinc treatment of childhood diarrhea in Bangladesh that included private-sector participation presents a promising approach for scale-up of CHoBI7 intervention program components [31]. Another suggestion was to have a demonstration handwashing station and drinking water container on display in health facilities for patients and family members to use and for health providers to recommend their purchase. Outside icddr,b’s hospital, shopkeepers sell items commonly used in the diarrhea ward (e.g. sippy cups and bedding). Partnerships with these individuals to sell CHoBI7 technologies could also be explored. In our recent RCT of the modified intervention (George et al., 2020 submitted), several neighbors of enrolled households made their own enabling technology using materials they obtained from the local market, which suggests that a model display in the hospital could facilitate uptake. Government stakeholder recommendations for integration of the CHoBI7 intervention program into the services provided in government *upazila* health complexes and community clinics are encouraging. Future research is needed to pilot the intervention program in these health facilities.

It is important to note that some recommendations made by government stakeholders were not possible to incorporate in this iteration of intervention adaptation (e.g. mass media campaign or selling of enabling technologies and supplies), given the intention to test the effectiveness of the intervention through an RCT. A key objective of this formative research was to define a more scalable approach for CHoBI7 program delivery. Developing a scalable intervention is an iterative process. The first iteration of the CHoBI7 intervention program was not scalable due to its intensive home visits. In the second iteration, presented here, we reduced the cost of enabling technologies, reduced the number of home visits in one arm, and eliminated home visits altogether in the mHealth with no home visits arm. We also added a mobile message component to reinforce the key behaviors promoted by the program over time. Our next step is to scale this program in partnership with the Ministry of Health and Family Welfare, incorporating the suggestions made by governmental stakeholders.

Mangham and Hanson identify four major challenges to scaling up interventions in international health: costs associated with scaling up coverage, scaling constraints, concerns for equity and quality, and issues related to service delivery [32]. Challenges with the reach, sustainability, and scalability of the CHoBI7 intervention program remain. First, a challenge to delivering the CHoBI7 intervention program in government health facilities is variability in the availability of WASH infrastructure, such as running water. Second, human resources in government health facilities in Bangladesh are often limited [33]. Third, in Bangladesh, informal health providers, including village doctors and pharmacists, may be visited before care is sought at a health facility [34, 35]. Future studies should assess approaches to include informal health providers.

The formative research presented here includes several strengths. One strength of the design was the involvement of pilot study participants as consultants to improve the intervention. This practice follows similar research [36] that suggests that the direct involvement of beneficiaries and an iterative process for intervention design comprise a valuable approach to tailoring interventions. A second strength was the iterative nature of the formative research, which allowed us to tailor the intervention as we learned and to explore emergent themes that further deepened our understanding of households’ experiences with the intervention and behavioral recommendations. Finally, our partnership with key government stakeholders allowed us to consider pathways to scale early in the process and develop support for a scalable approach to the intervention.

Diarrhea remains a top reason for seeking care at a health facility in Bangladesh [37]. There is a high risk of transmission of enteric disease within the health facility and home for household members of diarrhea patients [3–5, 7]. The CHoBI7 intervention program presents an important opportunity to intervene during this high-risk period, when perceived susceptibility of illness is high, perceived benefits of protective WASH behaviors are also likely to be high [18], and the environment is favorable for habit formation [19]. Other studies have also provided evidence that WASH interventions in health facilities can facilitate sustained behavior change [12, 15]. The CHoBI7 intervention program differs from more traditional, community-based WASH interventions, given that it is health facility-initiated and facilitates changes in behavior both in the health facility and once the patient and caregivers return home. Future work should prioritize WASH interventions for those at high risk of enteric diseases that can be taken to scale in health facilities—and sustained in households—to reduce future enteric disease episodes.

One limitation is that our interviews with pilot study participants were mostly with women. Although much of this imbalance was due to the availability of participants, the interviews we were able to conduct with pilot household men helped us to better understand how to incorporate their needs and schedules into our behavioral recommendations. Additional interviews with men may provide important insight into how to better balance household responsibilities related to WASH behaviors. Finally, future formative research should include interviews with health facility staff at multiple levels of care and management (e.g. doctors, nurses, and janitorial staff) to better inform our understanding of the practices and resources in health facilities that may impact delivery of the CHoBI7 intervention program.

## Conclusion

Formative research identified existing WASH and diarrhea patient care practices, target population experiences with and acceptability of a health facility-initiated WASH intervention, and facilitated modifications to the CHoBI7 intervention program in an effort to identify a scalable approach to deliver this program in Bangladesh.

## Data Availability

The datasets generated and/or analyzed during the current study are not publicly available due to identifiable content.

## Abbreviations

CHoBI7: Cholera-Hospital-based-Intervention-for-7-Days
WASH: Water, sanitation, and hygiene
IBM-WASH: Integrated Behavioural Model for Water, Sanitation, and Hygiene
RCT: Randomized controlled trial
ORS: Oral rehydration solution
icddr,b: International Centre for Diarrhoeal Disease Research, Bangladesh
NGOs: Non-Governmental Organizations
CDC: Communicable Diseases Control
